# Meningitis Mortality in U.S. Adults Aged ≥25 Years: Demographic and Geographic Insights from the CDC WONDER Database (1999–2024)

**DOI:** 10.3390/pathogens15030331

**Published:** 2026-03-19

**Authors:** Hassaan Abid, Muhammad Jawad, Muhammad Vazaym, Gaaitri Lohano, Syed Mohamin Abbas Shah, Naveed Ahmed Khan, Abdullah Afridi, Muhammad Mohid Haroon

**Affiliations:** 1 Department of Internal Medicine, Indiana University School of Medicine, Indianapolis, IN 46202, USA; 2 Department of Internal Medicine, Liaquat University of Medical and Health Sciences, Jamshoro 76090, Pakistan; shaikhjawad548@gmail.com (M.J.); vazaymmac15@gmail.com (M.V.); gaitrilohano@gmail.com (G.L.); 3 Department of Internal Medicine, King Edward Medical University, Lahore 54000, Pakistan; mohaminabbas@kemu.edu.pk; 4 Department of Internal Medicine, Khyber Medical College, Peshawar 25120, Pakistan; az862269@gmail.com (N.A.K.); abdullah7@kmc.edu.pk (A.A.); 5 Department of Internal Medicine, Ameer-ud-Din Medical College, Lahore 54000, Pakistan; mohidharoon123@gmail.com

**Keywords:** meningitis, mortality trends, CDC WONDER, epidemiology, demographic disparities, geographic variation

## Abstract

Meningitis remains a significant cause of morbidity and mortality in the United States despite advances in vaccination, antimicrobial therapy, and critical care. However, long-term national mortality patterns across demographic and geographic subgroups remain incompletely characterized. This study evaluated temporal trends in meningitis-associated mortality among U.S. adults aged ≥25 years from 1999 to 2024 using the Centers for Disease Control and Prevention Wide-Ranging Online Data for Epidemiologic Research (CDC WONDER) Multiple Cause of Death database. Death certificates listing meningitis as either the underlying cause or a contributing cause of death were identified using ICD-10 codes to capture meningitis-associated mortality. Age-adjusted mortality rates (AAMRs) per 100,000 population were calculated using the 2000 U.S. standard population. Temporal trends were assessed using Joinpoint regression to estimate annual percent change (APC) and average annual percent change (AAPC). Across the study period, meningitis-associated mortality demonstrated an early decline followed by stabilization and more recent increases in several subgroups. AAMRs decreased significantly from 1999 to 2001 (APC: −14.09%; *p* = 0.0029) and from 2001 to 2013 (APC: −4.74%; *p* < 0.000001), followed by a significant increase from 2013 to 2024 (APC: 1.78%; *p* = 0.0059). Despite these later increases, the overall AAPC across the full study period remained significantly negative (AAPC: −2.73%; *p* < 0.000001). Earlier analyses using shorter observation windows did not demonstrate a statistically significant overall trend; the significance observed in the present analysis reflects the inclusion of extended mortality data through 2024. Mortality rates were consistently higher among males and adults aged ≥65 years. Non-Hispanic Black individuals experienced increasing mortality after 2013, while Hispanic individuals demonstrated a sustained increase across the entire study period. Regional analyses showed recent increases in the Northeast, South, and West, while non-metropolitan areas did not experience statistically significant improvement through 2020. In summary, meningitis-associated mortality among U.S. adults declined significantly during the early study years but has shown stabilization and recent increases in several demographic and geographic subgroups since approximately 2013. Although overall mortality across the full study period remains lower than baseline levels, these emerging disparities highlight the importance of continued surveillance and targeted public health interventions.

## 1. Introduction

Meningitis is an inflammatory condition of the meninges, the protective membranes surrounding the brain and spinal cord, most commonly caused by bacterial, viral, fungal, or parasitic pathogens [1,2]. The clinical presentation ranges from mild, self-limited illness to rapidly progressive, life-threatening disease, depending on the causative organism, host factors, and timeliness of treatment. Despite substantial advances in vaccination strategies, antimicrobial therapy, and supportive care, meningitis remains a serious public health concern in the United States because of its potential for rapid progression, high fatality rates, and long-term neurological sequelae [1,2].

Among the various etiologies, bacterial meningitis is associated with the greatest morbidity and mortality and may result in severe complications such as septic shock, disseminated intravascular coagulation, elevated intracranial pressure, permanent neurological injury, and death [1,2]. Current estimates suggest an annual incidence of approximately 0.9 cases per 100,000 population in the United States, with case fatality rates exceeding 14%. Mortality risk is particularly elevated among older adults, individuals with compromised immune systems, and those with chronic medical conditions, reflecting both increased susceptibility and reduced physiological reserve [1]. Even with prompt treatment, survivors of bacterial meningitis may experience long-term consequences, including hearing loss, cognitive impairment, seizures, and motor dysfunction, imposing substantial individual and societal burdens [3].

The epidemiology of meningitis has evolved over recent decades, largely driven by the widespread introduction of conjugate vaccines targeting *Haemophilus influenzae* type b, *Streptococcus pneumoniae*, and *Neisseria meningitidis* [4,5]. These vaccination programs have contributed to substantial reductions in meningitis incidence and mortality, particularly among children [5]. However, meningitis-associated deaths continue to occur, and the burden of disease has increasingly shifted toward older age groups [6]. Adults aged 65 years and older experience disproportionately higher mortality rates, a pattern attributed to population aging, increased prevalence of comorbid conditions, and differences in pathogen distribution compared with younger populations [6].

In addition to demographic shifts, emerging challenges may contribute to persistent meningitis mortality. These include changes in circulating bacterial serotypes following vaccine introduction, the emergence of antimicrobial resistance, and increasing recognition of invasive fungal meningitis among immunocompromised individuals [7]. Fungal meningitis, although less common than bacterial or viral forms, is associated with high mortality and diagnostic delays, particularly in patients with HIV infection, malignancy, or those receiving immunosuppressive therapies [8]. Collectively, these factors underscore the importance of continued surveillance and careful evaluation of meningitis-associated mortality trends.

Understanding long-term patterns in meningitis mortality is important for assessing the public health impact of prevention strategies, identifying high-risk populations, and guiding future interventions. The Centers for Disease Control and Prevention’s Wide-Ranging Online Data for Epidemiologic Research (CDC WONDER) database compiles death certificate–based mortality data across the United States, enabling analyses by year, age, sex, and geographic region. Using CDC WONDER data, this study examines meningitis-associated mortality trends among U.S. adults aged ≥25 years from 1999 through 2024, with the aim of characterizing temporal changes and informing ongoing public health efforts.

## 2. Materials and Methods

### 2.1. Study Setting

For this database study, death certificate data were retrieved from the Centers for Disease Control and Prevention Wide-Ranging Online Data for Epidemiologic Research (CDC WONDER) Multiple Cause of Death database and examined from 1999 to 2024 to evaluate meningitis-associated mortality among adults aged ≥25 years in the United States. Cases were identified using the International Statistical Classification of Diseases and Related Health Problems, Tenth Revision (ICD-10) codes corresponding to meningitis diagnoses, including A87 (viral meningitis), G00 (bacterial meningitis, not elsewhere classified), A32.1 (listerial meningitis and meningoencephalitis), A39.0 (meningococcal meningitis), A17.0 (tuberculous meningitis), B00.3 (herpesviral meningitis), B01.0 (varicella meningitis), B02.1 (zoster meningitis), B37.5 (candidal meningitis), B45.1 (cerebral cryptococcosis), B38.4 (coccidioidomycosis meningitis), and G03 (meningitis due to other and unspecified causes). To enhance diagnostic specificity, meningococcal infection codes were restricted to A39.0 rather than broader three-digit A39 codes, thereby minimizing inclusion of non-meningitis conditions such as meningococcemia. The G00 category includes organism-specific bacterial meningitis subcodes such as pneumococcal (G00.1), *Haemophilus influenzae* (G00.0), group B streptococcal (G00.3), and other bacterial etiologies, thereby capturing major bacterial causes referenced in prior literature.

Death certificates listing meningitis as either the underlying cause of death or a contributing cause of death were included. This approach captures meningitis-associated mortality rather than deaths directly attributable to meningitis alone, because meningitis may contribute to death through complications such as sepsis, neurological injury, or systemic infection even when another condition is recorded as the primary cause on the death certificate. The use of multiple-cause mortality data is commonly applied in CDC WONDER analyses to better characterize the broader population burden of infectious diseases. Because both underlying and contributing causes were included, the findings should be interpreted as trends in meningitis-associated mortality rather than deaths attributable solely to meningitis as the underlying cause.

Institutional review board approval was not required because this study utilized deidentified, publicly available data. All research procedures adhered to the Strengthening the Reporting of Observational Studies in Epidemiology (STROBE) guidelines [9].

### 2.2. Data Extraction

The dataset was analyzed for demographic and geographic variables, including sex, ethnicity, age group, U.S. Census region, state, and urban–rural classification. Ethnicity (as defined in CDC WONDER death certificate data) was categorized as non-Hispanic White, non-Hispanic Black or African American, non-Hispanic Asian or Pacific Islander, non-Hispanic American Indian or Alaska Native, and Hispanic or Latino. Age was stratified into the following categories: 25–34, 35–44, 45–54, 55–64, 65–74, 75–84, and ≥85 years. For selected trend analyses, age groups were additionally collapsed into <65 years and ≥65 years to facilitate comparison between younger and older adult populations.

Urban–rural classification followed the 2013 National Center for Health Statistics Urban–Rural Classification Scheme, in which urban areas included large metropolitan areas (population ≥ 1 million) and medium or small metropolitan areas (population 50,000–999,999), while rural areas included counties with populations < 50,000 [10]. Regions were classified according to U.S. Census Bureau definitions as Northeast, Midwest, South, and West. The Northeast includes CT, ME, MA, NH, RI, VT, NJ, NY, and PA; the Midwest includes IL, IN, MI, OH, WI, IA, KS, MN, MO, NE, ND, and SD; the South includes DE, FL, GA, MD, NC, SC, VA, DC, WV, AL, KY, MS, TN, AR, LA, OK, and TX; and the West includes AZ, CO, ID, MT, NV, NM, UT, WY, AK, CA, HI, OR, and WA.

### 2.3. Statistical Analysis

Age-adjusted mortality rates (AAMRs) per 100,000 population were calculated for meningitis-associated mortality. AAMRs were computed using the direct age-standardization method based on the 2000 U.S. standard population to account for differences in age distribution [11].

Temporal trends in AAMRs from 1999 to 2024 were analyzed using the Joinpoint Regression Program (version 4.9.0.0, National Cancer Institute), which fits log-linear regression models to identify statistically significant changes in trend. The Joinpoint regression model employed permutation tests with standard error adjustments as implemented in the National Cancer Institute software, which account for potential autocorrelation in time-series trend estimation.

Annual percent change (APC) was calculated for each identified segment, and average annual percent change (AAPC) was calculated for the full study period. APC and AAPC values were considered statistically significant if the two-sided *p*-value was <0.05.

Given the number of subgroup analyses performed, findings should be interpreted with caution due to the potential for type I error arising from multiple comparisons. No formal adjustment for multiple testing was applied; therefore, subgroup findings should be interpreted cautiously and are considered exploratory.

The analysis period was extended to 2024 using the most recent CDC WONDER mortality release to provide an updated evaluation of long-term trends. Earlier analyses based on shorter observation windows demonstrated similar directional patterns but did not reach statistical significance for the full-period trend estimate. To evaluate whether inclusion of contributing causes of death influenced the observed temporal trends, a sensitivity analysis was conducted, restricting the dataset to deaths in which meningitis was recorded as the underlying cause of death (UCD). Joinpoint regression was repeated using the same analytical framework to compare temporal patterns with the primary analysis based on meningitis-associated mortality. 

The extracted data and the joinpoint graphs of the analysis are available in the Supplementary Materials section. 

## 3. Results

### 3.1. Overall

**Figure 1:**
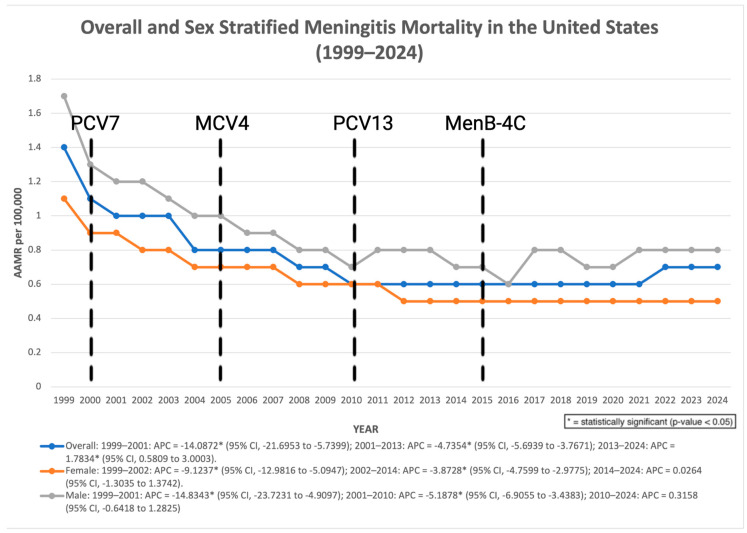
Overall and sex-stratified AAMR per 100,000 in the United States, 1999–2024. Dashed vertical lines indicate approximate years of major vaccine introductions, including pneumococcal conjugate vaccines (2000, 2010), meningococcal conjugate vaccine (2005), and meningococcal B vaccine (2015).

**Figure 2:**
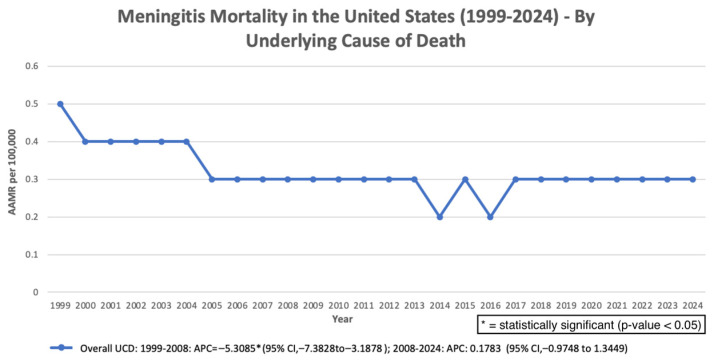
Overall AAMR per 100,000 with meningitis as the underlying cause of death in the United States, 1999–2024.

Across the study period (1999–2024), meningitis-associated mortality demonstrated an initial period of significant decline followed by a later increase. The age-adjusted mortality rate (AAMR) decreased significantly from 1999 to 2001 (APC: −14.09%; 95% CI: −21.70 to −5.74; *p* = 0.0029), and continued to decline from 2001 to 2013 (APC: −4.74%; 95% CI: −5.69 to −3.77; *p* < 0.000001). However, from 2013 to 2024, mortality increased significantly (APC: 1.78%; 95% CI: 0.58 to 3.00; *p* = 0.0059). Despite this recent increase, the overall AAPC across the full study period remained significantly negative (AAPC: −2.73%; 95% CI: −3.66 to −1.79; *p* < 0.000001) (Figure 1). Earlier analyses using shorter follow-up periods showed similar directional patterns but did not reach statistical significance for the overall trend estimate. In sensitivity analyses restricted to deaths in which meningitis was recorded as the underlying cause of death, similar temporal patterns were observed. Mortality declined significantly from 1999 to 2008, followed by a non-significant change from 2008 to 2024. Across the full study period, the overall trend remained significantly negative (AAPC −1.83%, *p* = 0.0004), supporting the robustness of the primary findings (Figure 2).

### 3.2. Gender

Throughout the study period, males consistently exhibited higher mortality rates than females.

Among males, mortality declined significantly from 1999 to 2001 (APC: −14.83%; 95% CI: −23.72 to −4.90; *p* = 0.0067), followed by a further significant decrease from 2001 to 2010 (APC: −5.19%; 95% CI: −6.90 to −3.44; *p* = 0.000009). From 2010 to 2024, the trend stabilized and was not statistically significant (APC: 0.32%; 95% CI: −0.64 to 1.28; *p* = 0.4986). Overall, males experienced a significant decline in mortality across the study period (AAPC: −2.98%; 95% CI: −4.08 to −1.87; *p* < 0.000001). Among females, mortality declined significantly from 1999 to 2002 (APC: −9.12%; 95% CI: −12.98 to −5.10; *p* = 0.0002), followed by a continued significant decline between 2002 and 2014 (APC: −3.87%; 95% CI: −4.75 to −2.98; *p* < 0.000001). From 2014 to 2024, mortality remained stable, with no statistically significant change (APC: 0.03%; 95% CI: −1.30 to 1.37; *p* = 0.9674). Across the entire study period, females also experienced a significant overall decline (AAPC: −2.99%; 95% CI: −3.77 to −2.20; *p* < 0.000001) (Figure 1).

### 3.3. Ethnicity

**Figure 3:**
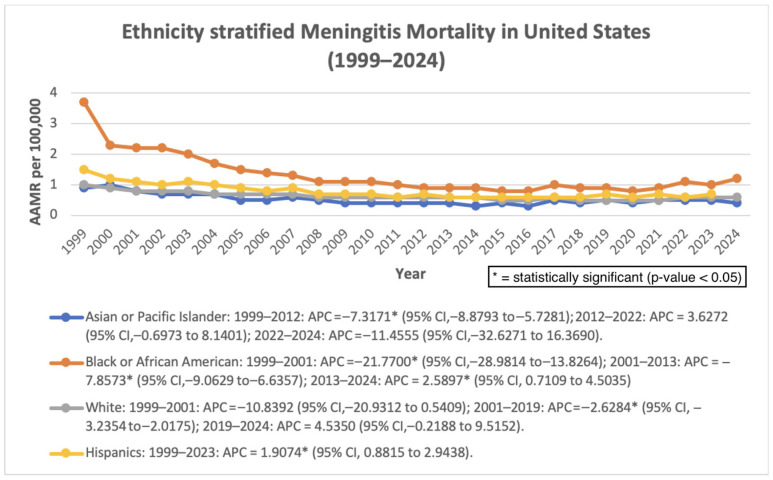
Ethnicity-stratified AAMR per 100,000 in the United States, 1999–2024.

Among Asian or Pacific Islander individuals, mortality declined significantly from 1999 to 2012 (APC: −7.32%; 95% CI: −8.88 to −5.73; *p* < 0.000001). From 2012 to 2022, mortality demonstrated a non-significant increase (APC: 3.63%; 95% CI: −0.70 to 8.14; *p* = 0.0961), followed by a non-significant decline between 2022 and 2024 (APC: −11.46%; 95% CI: −32.63 to 16.37; *p* = 0.3620). Overall, mortality declined significantly across the study period (AAPC: −3.44%; 95% CI: −6.03 to −0.78; *p* = 0.0115). Among Black or African American individuals, mortality decreased significantly from 1999 to 2001 (APC: −21.77%; 95% CI: −28.98 to −13.83; *p* = 0.000045), followed by continued decline from 2001 to 2013 (APC: −7.86%; 95% CI: −9.06 to −6.64; *p* < 0.000001). From 2013 to 2024, mortality increased significantly (APC: 2.59%; 95% CI: 0.71 to 4.50; *p* = 0.0094). Despite this recent increase, the overall trend remained significantly decreasing (AAPC: −4.66%; 95% CI: −5.79 to −3.50; *p* < 0.000001). Among White individuals, mortality showed a non-significant decline between 1999 and 2001 (APC: −10.84%; 95% CI: −20.93 to 0.54; *p* = 0.0601), followed by a significant decrease from 2001 to 2019 (APC: −2.63%; 95% CI: −3.24 to −2.02; *p* < 0.000001). Between 2019 and 2024, mortality increased non-significantly (APC: 4.54%; 95% CI: −0.22 to 9.52; *p* = 0.0606). Overall, mortality declined significantly across the full study period (AAPC: −1.93%; 95% CI: −3.21 to −0.63; *p* = 0.0037). Among Hispanic individuals, mortality demonstrated a consistent and statistically significant increase from 1999 to 2024 (AAPC: 1.90%; 95% CI: 0.88 to 2.94; *p* = 0.0008). Across the study period, non-Hispanic Black individuals consistently demonstrated the highest AAMRs, followed by Hispanic and non-Hispanic White populations. (Figure 3).

### 3.4. Age Group

For trend analysis, age groups were collapsed into <65 years and ≥65 years.

**Figure 4:**
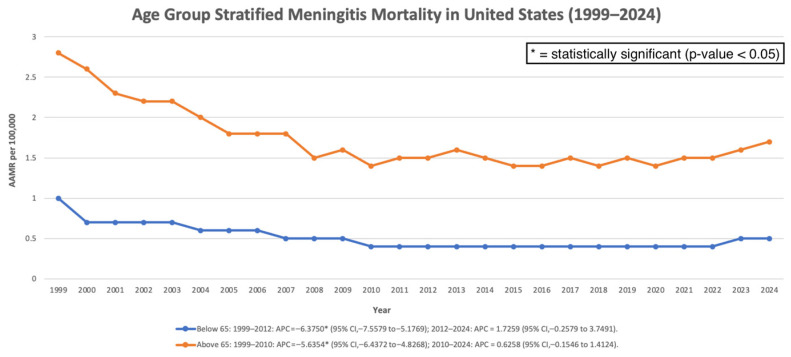
Age-stratified AAMR per 100,000 in the United States, 1999–2024.

Among individuals younger than 65 years, mortality declined significantly from 1999 to 2012 (APC: −6.38%; 95% CI: −7.56 to −5.18; *p* < 0.000001). From 2012 to 2024, mortality showed a non-significant increase (APC: 1.73%; 95% CI: −0.26 to 3.75; *p* = 0.085). Overall, the full-period trend remained significantly decreasing (AAPC: −2.57%; 95% CI: −3.62 to −1.51; *p* = 0.000003). Among individuals aged ≥65 years, mortality decreased significantly between 1999 and 2010 (APC: −5.64%; 95% CI: −6.44 to −4.83; *p* < 0.000001). From 2010 to 2024, mortality demonstrated a slight but non-significant increase (APC: 0.63%; 95% CI: −0.15 to 1.41; *p* = 0.110). Across the full study period, the AAPC remained significantly negative (−2.18%; 95% CI: −2.71 to −1.65; *p* < 0.000001) (Figure 4).

### 3.5. Geographical Regions

All regions demonstrated early declines followed by stabilization or increases in later years.

**Figure 5:**
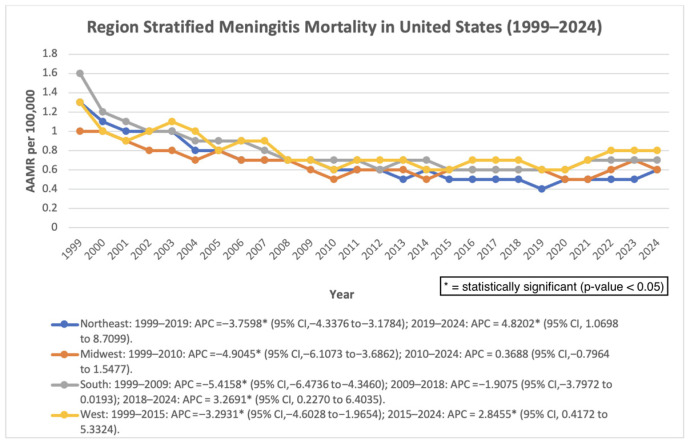
Census region-stratified AAMR per 100,000 in the United States, 1999–2024.

The Northeast experienced a significant decline from 1999 to 2019 (APC: −3.76%; *p* < 0.000001), followed by a significant increase from 2019 to 2024 (APC: 4.82%; *p* = 0.0138). The overall AAPC remained significantly negative (−2.10%; *p* < 0.000001). The Midwest showed a significant decline from 1999 to 2010 (APC: −4.90%; *p* < 0.000001) and stable trends thereafter (AAPC: −1.99%; *p* = 0.000001). The South declined significantly from 1999 to 2009 (APC: −5.42%; *p* < 0.000001), followed by a significant increase from 2018 to 2024 (APC: 3.27%; *p* = 0.0364). Overall, AAPC remained negative (−2.13%; *p* = 0.000040). The West declined significantly from 1999 to 2015 (APC: −3.29%; *p* = 0.000047) but increased significantly from 2015 to 2024 (APC: 2.85%; *p* = 0.0235). The overall AAPC was not statistically significant (−1.13%; *p* = 0.0545) (Figure 5).

### 3.6. Urbanization

**Figure 6:**
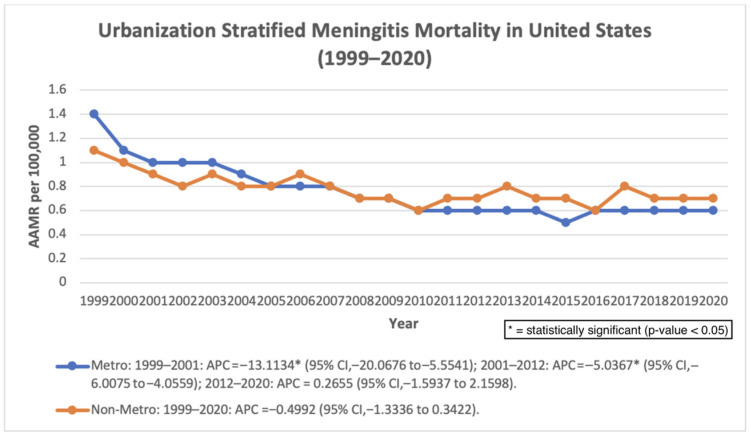
Urbanization-stratified AAMR per 100,000 in the United States, 1999–2020.

Urban–rural classification data were available through 2020. In metropolitan areas, mortality declined significantly from 1999 to 2001 (APC: −13.11%; *p* = 0.0028) and from 2001 to 2012 (APC: −5.04%; *p* < 0.000001), followed by a non-significant change through 2020. The overall AAPC remained significantly negative (−3.87%; *p* < 0.000001). In non-metropolitan areas, mortality showed a non-significant decline from 1999 to 2020 (AAPC: −0.50%; *p* = 0.2294) (Figure 6).

### 3.7. Stratified by State

**Figure 7:**
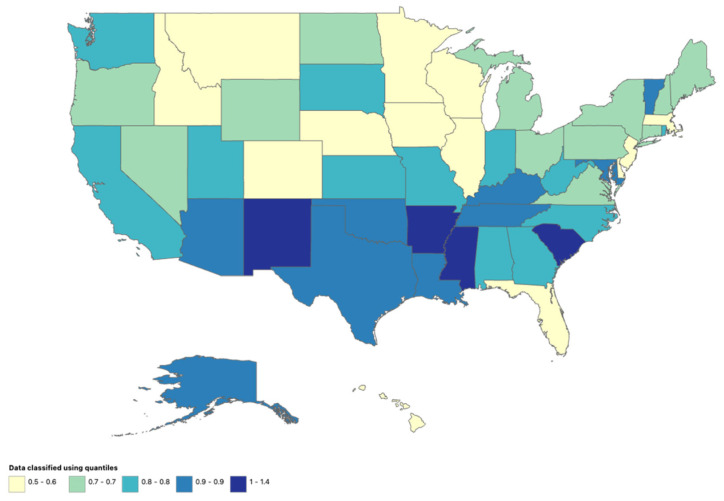
State-stratified AAMR per 100,000 in the United States, 1999–2020.

State-level analyses were available for 1999–2020. During this period, a total of 34,786 meningitis-associated deaths were recorded among adults aged ≥25 years in the United States. The highest number of deaths occurred in California (4249), followed by Texas (2859), New York (2178), Florida (1731), and Pennsylvania (1520). The District of Columbia recorded the highest age-adjusted mortality rate (AAMR: 1.4 per 100,000). Several states demonstrated elevated AAMRs of 1.0, including Arkansas, Mississippi, New Mexico, and South Carolina (Figure 7).

### 3.8. Stratified by Etiology

For bacterial meningitis, mortality declined significantly from 1999 to 2001 (APC: −13.74%; *p* < 0.000001), followed by fluctuations and relative stability after 2009, with a very small yet statistically significant increase from 2009 to 2024 (APC: 0.03%; *p* < 0.000001), indicating minimal absolute change despite statistical significance. Overall, the AAPC remained significantly negative (−2.61%; *p* < 0.000001).

**Figure 8:**
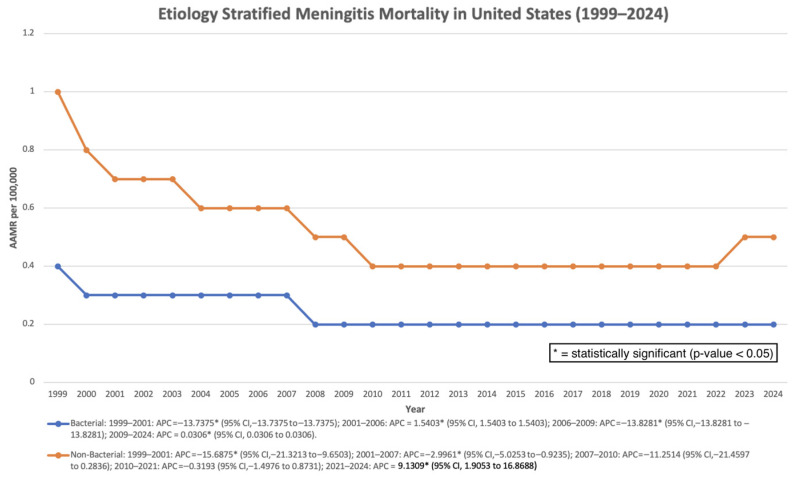
Etiology-stratified mortality rate per 100,000 in the United States, 1999–2024.

For non-bacterial meningitis, mortality declined significantly in early years, remained stable through 2021, and increased significantly between 2021 and 2024 (APC: 9.13%; *p* = 0.0167). Despite this recent rise, the overall AAPC remained significantly negative (−2.59%; *p* = 0.0029) (Figure 8).

## 4. Discussion

This nationwide analysis of CDC WONDER mortality data from 1999 to 2024 demonstrates that meningitis-associated mortality among U.S. adults aged ≥25 years followed a non-linear pattern characterized by significant early declines followed by stabilization and more recent increases in several populations. Joinpoint regression identified significant decreases in age-adjusted mortality rates between 1999 and 2013, followed by a statistically significant increase from 2013 to 2024. Although the overall average annual percent change (AAPC) across the entire study period remained significantly negative, this estimate largely reflects the magnitude of earlier declines rather than a uniform decrease across the entire time frame. Earlier analyses using shorter observation windows did not demonstrate a statistically significant overall trend; the significance observed in the present analysis reflects the inclusion of extended mortality data through 2024.

Interpretation of long-term trend estimates should therefore consider the observation window used for analysis. Earlier analyses using shorter follow-up periods demonstrated similar directional patterns but did not always reach statistical significance for the full-period trend estimate. With the inclusion of more recent mortality data through 2024, the cumulative effect of sustained early declines resulted in a statistically significant overall AAPC. These findings therefore reflect updated trend estimates based on the extended dataset rather than evidence of a continuous decline across the entire study period. The more recent increases observed after approximately 2013 suggest that earlier improvements in meningitis-associated mortality may not have been sustained uniformly. Sensitivity analyses restricted to deaths in which meningitis was recorded as the underlying cause demonstrated comparable patterns, with significant declines during earlier years followed by stabilization in more recent years, and an overall negative AAPC across the full study period. These findings support the robustness of the primary results and suggest that the observed temporal trends are not solely driven by the inclusion of contributing causes of death in mortality classification.

The early reductions in mortality observed between 1999 and approximately 2013 likely reflect the cumulative impact of several public health interventions. Widespread implementation of conjugate vaccines targeting *Haemophilus influenzae* type b, *Streptococcus pneumoniae*, and *Neisseria meningitidis* has substantially reduced the incidence of invasive bacterial meningitis, particularly among younger populations [12,13,14]. Improvements in antimicrobial therapy, rapid diagnostic methods, and advances in intensive care management have also contributed to improved survival among patients with severe infections. Enhanced laboratory diagnostics, including polymerase chain reaction-based testing and more sensitive cerebrospinal fluid analysis techniques, may have facilitated earlier pathogen identification and treatment initiation.

Despite these early improvements, the recent increases observed in several subgroups suggest evolving epidemiologic dynamics. Changes in circulating pathogen serotypes following vaccine introduction, the emergence of antimicrobial resistance, and demographic shifts toward an aging population may contribute to increased vulnerability to severe infections [15,16,17]. Additionally, a growing population of immunocompromised individuals due to malignancy, organ transplantation, immunosuppressive therapies, and HIV infection may increase susceptibility to invasive infections, including fungal meningitis [18,19]. These factors may partially explain the stabilization or increases observed in recent years.

Sex-based differences persisted throughout the study period, with males consistently demonstrating higher mortality rates than females. These findings are consistent with prior studies suggesting biological and behavioral differences in susceptibility to infectious diseases. Sex-related variations in immune response, comorbidity profiles, occupational exposures, and healthcare utilization patterns may contribute to these disparities [18,19,20]. Although both males and females experienced significant overall declines during earlier years, the absence of continued improvement in recent years indicates that sex-based disparities remain present.

Racial and ethnic disparities in meningitis-associated mortality were also observed. Non-Hispanic Black individuals experienced higher mortality rates throughout the study period and demonstrated increasing mortality after 2013, indicating a potential reversal of earlier improvements. Hispanic individuals exhibited a sustained increase in mortality across the entire study period, contrasting with the declining trends observed in other populations. These disparities likely reflect complex interactions between socioeconomic factors, access to healthcare services, vaccination coverage, underlying health conditions, and structural determinants of health [21,22]. Differences in healthcare access, delays in diagnosis, and variations in preventive care utilization may contribute to these persistent inequities.

Age-stratified analyses demonstrated that both younger adults and those aged ≥65 years experienced significant early declines in mortality. However, trends stabilized or increased modestly in both groups in later years. Older adults remain particularly vulnerable to meningitis-associated mortality due to immunosenescence, higher prevalence of chronic medical conditions, and increased susceptibility to invasive infections [23,24]. The absence of sustained improvement in older populations highlights the need for prevention strategies that specifically address the risks associated with aging populations.

Etiology-specific analyses demonstrated differing temporal patterns between bacterial and non-bacterial meningitis. Mortality from bacterial meningitis declined during earlier years and remained relatively stable thereafter, whereas non-bacterial meningitis demonstrated a statistically significant increase in the most recent years of the study period. These changes may reflect shifts in pathogen epidemiology, improved recognition of fungal meningitis in immunocompromised populations, or evolving diagnostic and reporting practices rather than a single causal factor [25].

Geographic analyses revealed notable regional heterogeneity. While all U.S. Census regions experienced early declines in mortality, the Northeast, South, and West demonstrated statistically significant increases in more recent years. These findings suggest that regional differences in population demographics, healthcare infrastructure, vaccination uptake, and public health resources may influence mortality trajectories [26]. State-level analyses further demonstrated substantial variability, with several states and the District of Columbia exhibiting comparatively higher age-adjusted mortality rates.

Urban–rural comparisons demonstrated divergent patterns in mortality trends. Metropolitan areas experienced significant declines during earlier years, whereas non-metropolitan areas did not demonstrate statistically significant improvement during the available study period. These findings may reflect structural barriers to timely diagnosis and specialized care in rural settings. Limited access to infectious disease specialists, longer transport times to tertiary care centers, and reduced healthcare resources in rural hospitals may contribute to delayed recognition and treatment of severe infections [27,28].

It is important to note that this study evaluated meningitis-associated mortality, defined as deaths in which meningitis was recorded as either the underlying cause or a contributing cause on death certificates. This approach is commonly used in analyses of infectious disease mortality because meningitis may contribute to death through complications such as sepsis, neurologic injury, or systemic infection, even when another condition is listed as the primary cause. Consequently, the results reflect the broader population burden of meningitis-associated mortality rather than deaths attributable solely to meningitis as the underlying cause. This distinction is important when interpreting long-term mortality trends because changes in diagnostic recognition or death certification practices may influence whether meningitis is recorded as an underlying or contributing cause of death.

Finally, several subgroup analyses were conducted to explore demographic and geographic patterns in mortality trends. Because multiple comparisons increase the possibility of type I error, these subgroup findings should be interpreted cautiously and considered exploratory. Because numerous subgroup APC and AAPC estimates were evaluated, some statistically significant findings may also reflect random variation rather than true epidemiologic differences.

These findings highlight the importance of sustained national surveillance to monitor evolving meningitis epidemiology and to guide targeted prevention strategies in vulnerable populations.

## 5. Limitations

This study has several limitations. Mortality data derived from death certificates may be subject to misclassification or coding inaccuracies, and variations in diagnostic and reporting practices over time may influence mortality classification. The CDC WONDER database provides aggregated data and lacks detailed clinical information, including pathogen confirmation, disease severity, vaccination status, comorbidities, and antimicrobial resistance patterns. Socioeconomic factors and healthcare access variables were also unavailable. This analysis evaluated meningitis-associated mortality, defined as deaths in which meningitis was recorded as either the underlying or a contributing cause on the death certificate. While this approach captures the broader population burden of meningitis-related deaths, differences in death certification practices may influence classification. However, a sensitivity analysis restricted to deaths with meningitis as the underlying cause of death demonstrated similar temporal patterns, supporting the robustness of the findings. Finally, multiple subgroup analyses were performed, and no formal adjustment for multiple comparisons was applied; therefore, some statistically significant findings may reflect type I error. In addition, the ecological nature of aggregated mortality data precludes causal inference.

## 6. Clinical and Public Health Implications

Persistent demographic and geographic disparities in meningitis-associated mortality emphasize the importance of sustained vaccination efforts, improved access to timely diagnostic services, and targeted interventions for high-risk populations. The recent statistically significant increases observed in selected subgroups underscore the need for renewed surveillance and evaluation of emerging epidemiologic trends. Expanding healthcare access in underserved regions and strengthening infectious disease infrastructure in rural communities may contribute to reducing ongoing disparities [29,30,31,32].

## 7. Conclusions

In this nationwide analysis of U.S. adults aged ≥25 years, meningitis-associated mortality demonstrated a non-linear pattern characterized by significant declines during the early and mid-study years, followed by stabilization and more recent increases in several demographic and geographic subgroups after approximately 2013. While overall mortality across the full 1999–2024 period remains significantly lower than baseline levels, recent reversals in selected populations, particularly among Hispanic individuals and in certain regions, highlight emerging public health concerns. Continued surveillance, equity-focused prevention strategies, and sustained investment in infectious disease management are essential to prevent further erosion of prior gains.

## Data Availability

The datasets analyzed during the current study are publicly available at CDC WONDER: https://wonder.cdc.gov/ (accessed on 15 February 2026).

## Supplementary Materials

The following supporting information can be downloaded at: https://www.mdpi.com/article/10.3390/pathogens15030331/s1.

## Abbreviations

The following abbreviations are used in this manuscript:

AAPC	Average Annual Percent Change
APC	Annual Percent Change
AAMR	Age-Adjusted Mortality Rate
CDC	Centers for Disease Control and Prevention
CMR	Crude Mortality Rate
Hib	*Haemophilus influenzae* type b
ICD-10	International Statistical Classification of Diseases and Related Health Problems, 10th Revision
MenACWY	Quadrivalent Meningococcal Conjugate Vaccine (Serogroups A, C, W, Y)
MenB	Meningococcal Serogroup B Vaccine
PCV7	7-Valent Pneumococcal Conjugate Vaccine
PCV13	13-Valent Pneumococcal Conjugate Vaccine
STROBE	Strengthening the Reporting of Observational Studies in Epidemiology
WONDER	Wide-Ranging Online Data for Epidemiologic Research
