# Supplementary figures and images for: Meningitis Mortality in U.S. Adults Aged ≥25 Years: Demographic and Geographic Insights from the CDC WONDER Database (1999–2024)
Source: Pathogens. 2026 Mar 19;15(3):331. doi: 10.3390/pathogens15030331 (PMC13029061; doi:10.3390/pathogens15030331)

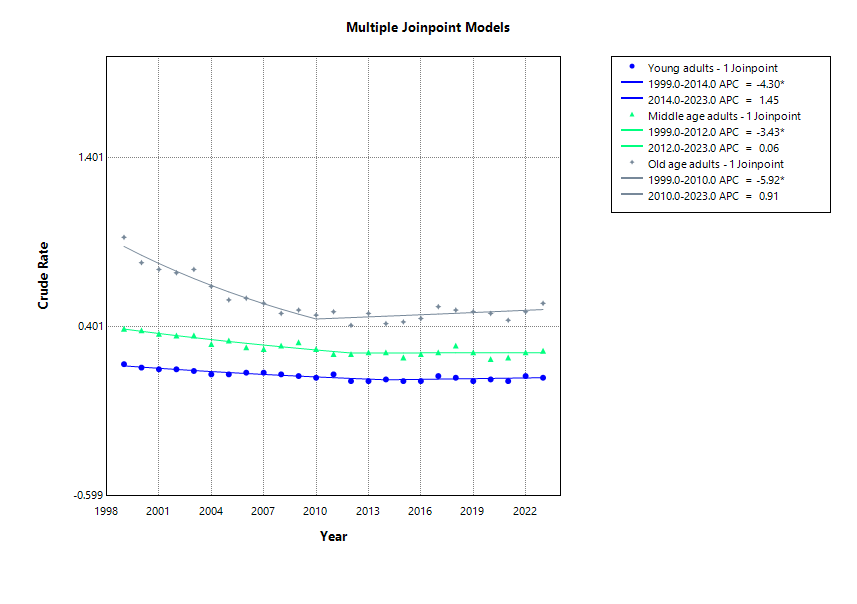

Supplement: Supplementary file 1 [file pathogens-15-00331-s001.zip › JP Graphs/Age.bmp]

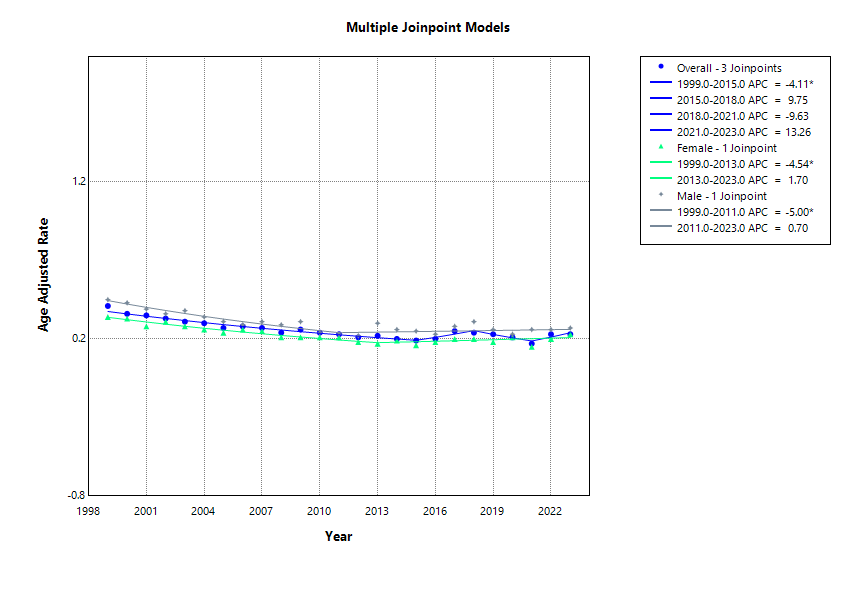

Supplement: Supplementary file 1 [file pathogens-15-00331-s001.zip › JP Graphs/Overall.bmp]

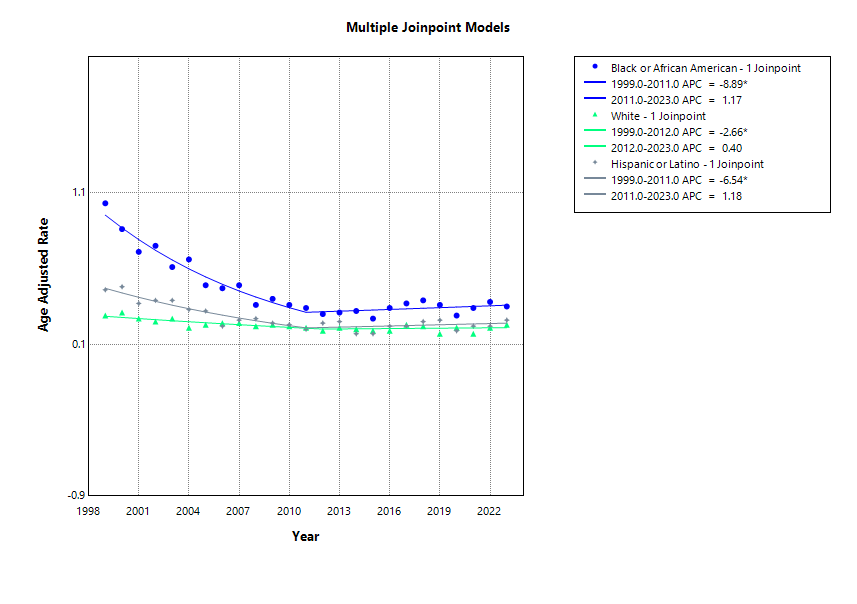

Supplement: Supplementary file 1 [file pathogens-15-00331-s001.zip › JP Graphs/Race.bmp]

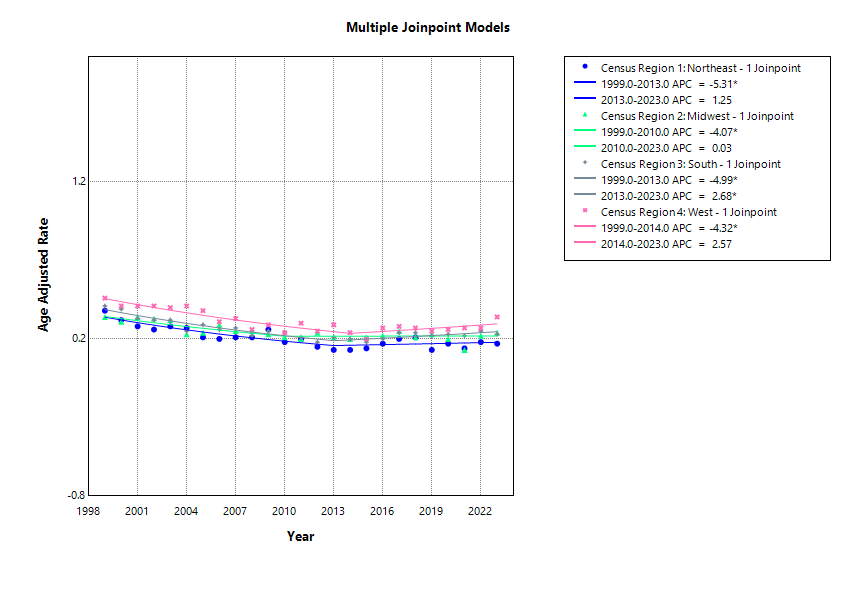

Supplement: Supplementary file 1 [file pathogens-15-00331-s001.zip › JP Graphs/Region.bmp]

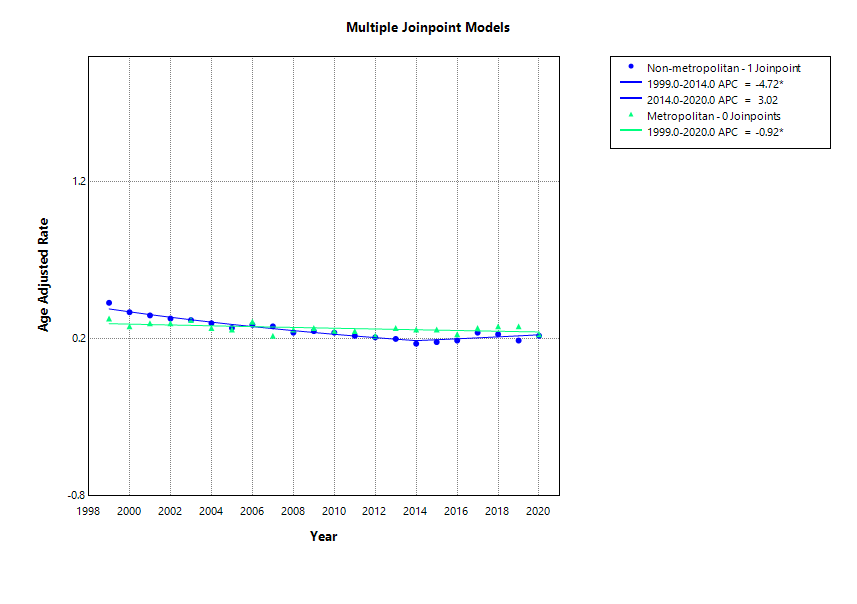

Supplement: Supplementary file 1 [file pathogens-15-00331-s001.zip › JP Graphs/Urbanization.bmp]
